# UCN-01 enhances cytotoxicity of irinotecan in colorectal cancer stem-like cells by impairing DNA damage response

**DOI:** 10.18632/oncotarget.9859

**Published:** 2016-06-06

**Authors:** Michele Signore, Mariachiara Buccarelli, Emanuela Pilozzi, Gabriele De Luca, Marianna Cappellari, Maurizio Fanciulli, Frauke Goeman, Elisa Melucci, Mauro Biffoni, Lucia Ricci-Vitiani

**Affiliations:** ^1^ Department of Hematology, Oncology and Molecular Medicine, Istituto Superiore di Sanità, Rome, Italy; ^2^ Department of Clinical and Molecular Medicine, Sant'Andrea Hospital, University La Sapienza, Rome, Italy; ^3^ Scientific Direction, Regina Elena National Cancer Institute, Rome, Italy

**Keywords:** colorectal cancer stem-like cells, kinase inhibitors, Chk1, DNA damage, phospho-proteomics

## Abstract

Colorectal cancer (CRC) is one of the most common and lethal cancers worldwide. Despite recent progress, the prognosis of advanced stage CRC remains poor, mainly because of cancer recurrence and metastasis. The high morbidity and mortality of CRC has been recently ascribed to a small population of tumor cells that hold the potential of tumor initiation, i.e. cancer stem cells (CSCs), which play a pivotal role in cancer recurrence and metastasis and are not eradicated by current therapy. We screened CRC-SCs *in vitro* with a library of protein kinase inhibitors and showed that CRC-SCs are resistant to specific inhibition of the major signaling pathways involved in cell survival and proliferation. Nonetheless, broad-spectrum inhibition by the staurosporin derivative UCN-01 blocks CRC-SC growth and potentiates the activity of irinotecan *in vitro* and *in vivo* CRC-SC-derived models. Reverse-Phase Protein Microarrays (RPPA) revealed that, albeit CRC-SCs display individual phospho-proteomic profiles, sensitivity of CRC-SCs to UCN-01 relies on the interference with the DNA damage response mediated by Chk1. Combination of LY2603618, a specific Chk1/2 inhibitor, with irinotecan resulted in a significant reduction of CRC-SC growth *in vivo*, confirming that irinotecan treatment coupled to inhibition of Chk1 represents a potentially effective therapeutic approach for CRC treatment.

## INTRODUCTION

Colorectal cancer (CRC) is the third most frequent cancer in both sexes and is also the second cause of cancer death in the western world [1]. CRC development results from a progressive transformation of colorectal epithelial cells following the accumulation of mutations in a number of oncogenes and tumor suppressor genes [2]. Despite progress in surgery, radiation and chemotherapy, none of the treatments available is curative for patients with advanced stage CRC. Therefore, developing new therapeutic strategies to eliminate tumor is ultimately critical. The current standard treatment for advanced and metastatic colorectal cancer is represented by the combination of 5-fluorouracil (5-FU) and folates with oxaliplatin (FOLFOX) or irinotecan (FOLFIRI) [3, 4], however the use of biologics directed to block some altered oncogenic pathways has also proven beneficial for a subset of patients. Cetuximab is an antibody that specifically blocks epidermal growth factor receptor (EGFR) oncogenic signaling in cancer cells. In association with conventional chemotherapy, Cetuximab has been shown to significantly increase the overall survival of patients with advanced colon cancer [5, 6]. Nonetheless almost 40% of CRC tumors bear KRAS-activating mutations and are refractory to Cetuximab treatment, indicating the relevance of genotyping tumors in order to select the most appropriate personalized treatment [7, 8].

The stepwise accumulation of specific genetic lesions is known to exert a driving force in tumor progression and oftentimes underlies the resistance or sensitivity to specific targeted drugs. Drug resistance is also associated with an increased efficiency of protective mechanisms, e.g. drug efflux channels or DNA repair machinery [9, 10], which usually define a subpopulation of cancer cells endowed with stem-like features and thus named cancer stem cells (CSCs). CSCs are responsible for tumor initiation and maintenance due to their intrinsic self-renewal capacity and the ability to differentiate, allowing the generation of a phenocopy of the original tumor upon injection into immunodeficient mice [11, 12]. Therefore, this subpopulation of cells represents a critical tool for the preclinical evaluation of new anticancer therapies. CRC cell lines have been widely used to investigate, both *in vitro* and *in vivo*, the genetic and epigenetic changes underlying tumor development as well as for drug screening and biomarker discovery studies [13–16]. In addition to the common advantages of commercially available colorectal cell lines, i.e. broad supply of live cells, ease of handling and controlling of experimental parameters, CRC-SC lines offer the unique opportunity to phenocopy the parental tumor and reproduce its transcriptional heterogeneity [17]. The establishment of improved preclinical models that recapitulate the human disease, preserving its cellular heterogeneity and histopathological or genetic alterations, is essential for testing the efficacy of new target-directed therapies and to identify drug response biomarkers [18]. In this study, we exploited two parallel experimental approaches, i.e. kinase inhibitor library screening and Reverse-Phase Protein Microarrays (RPPA) technology, to identify signaling pathways associated with the malignant behaviour of CRC-SC lines and evaluated the effects of interfering with them.

## RESULTS

### Isolation and characterization of colorectal cancer stem-like cell lines

Fifteen CRC-SC lines were obtained from colorectal adenocarcinoma patients. The main clinical characteristics of patients and the phenotypic features of the derived CRC-SC lines are listed in Supplementary Table S1. Tumor samples were subjected to mechanical and enzymatic dissociation and cultured in stem cell medium. All these CRC-SC lines were expanded *in vitro* and validated for their stem cell properties, by assessing the ability to self-renew, to generate progeny of multiple lineages in differentiating culture conditions and to faithfully reproduce patient's histology in mouse xenografts (Supplementary Figure S1). CRC-SC lines were also characterized for the expression of the stem cell marker CD133 and the epithelial marker Ber-Ep4 [12] (Supplementary Table S1). Their authenticity was evaluated by analysis of the short tandem repeat (STR) profile. Moreover, we performed targeted sequencing of 17 tumor-specific genes in all CRC-SC lines. The frequency of genetic alterations in our CRC-SC line samples confirms that they are representative of the CRC patient population (Table 1).

**Table 1:** Genetic alterations of 17 tumor-specific genes in CRC-SC lines

	1.1	1.2	18	383	385	389	393	398	416	417	430	432	85	CRO	438
ACVR1B			∎		∎				∎						
AMER1					∎				∎						∎
APC	∎	∎	∎	∎	∎		∎	∎	∎	∎	∎		∎		
BRAF									∎						∎
CTNNB1		∎		∎	∎		∎		∎			∎			
FBXW7			∎		∎	∎	∎				∎				
KIAA1804			∎						∎		∎				
KRAS			∎	∎		∎		∎				∎	∎		
MAP2K4					∎				∎						
NRAS	∎	∎							∎						
PIK3CA	∎	∎			∎	∎	∎	∎	∎	∎	∎				∎
PTEN			∎						∎				∎		
SMAD2				∎	∎				∎						∎
SMAD4	∎	∎				∎		∎	∎		∎		∎		
SOX9			∎			∎			∎						∎
TCF7L2	∎	∎	∎		∎				∎			∎			
TP53			∎	∎	∎	∎		∎	∎	∎		∎			

Criteria used to assigned mutations were: Allele Frequency >5%; SNP MAF <5%; Coverage >100x; Protein Damage Missense.

### CRC-SC lines display intrinsic resistance to small molecule kinase inhibitors

**Figure 1:**
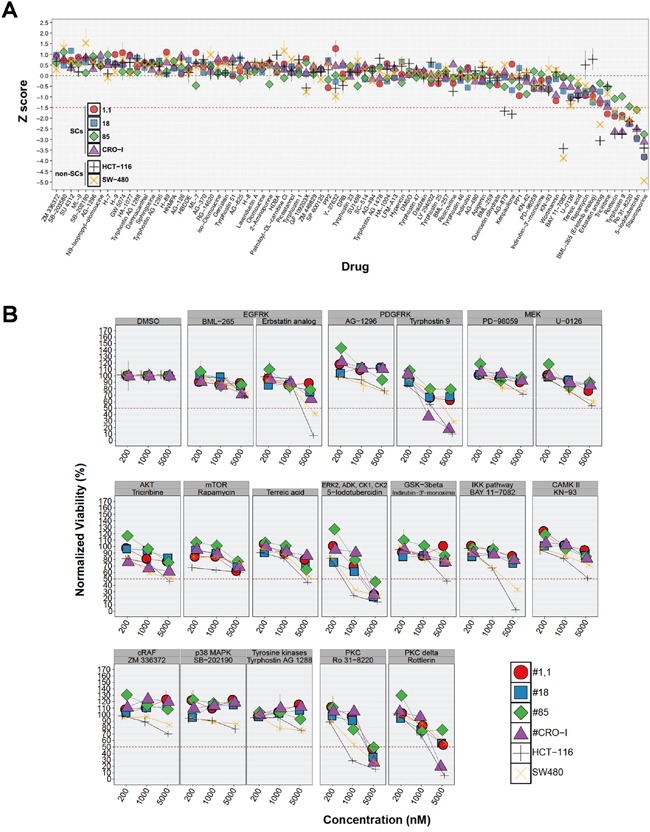
CRC-SC lines are resistant to drug-mediated inhibition of diverse signaling pathways **A.** For each non-SC and SC CRC-line results of drug response after 48h treatment with a small molecule kinase inhibitor library are reported as mean±SD (n=3) of standardized values (z score). Z scores were calculated from normalized viability values (V_D_, see Materials and Methods section) and the corresponding mean (mV_D_) and SD (sdV_D_) for each individual cell line using the following formula: z score=(V_D_–mV_D_)/sdV_D_. Dotted green and red horizontal lines denote the average (0) and −1.5 z scores, respectively. **B.** Titration of selected positive hits grouped by pathway target and other drugs included as experimental controls (individual drug names are indicated above each plot). Values are reported, for each cell line and drug tested, as the mean±SD of ≥2 independent experiments.

In order to identify molecular targets playing a major role in sustaining CRC-SC proliferation and survival, we first assessed the effect of a commercially available kinase inhibitor library on CRC-SC viability and/or proliferation. The drugs included in the screening library are low molecular weight protein kinase inhibitors (KI) targeting receptor tyrosine kinases (RTK), e.g. EGFR, and intracellular kinase pathways, e.g. PKC, considered important in cancer cell proliferation and/or survival. The complete list of the inhibitors and the pathways targeted is available in Supplementary Table S2. Staurosporine is the positive control of the library. Based on the knowledge in high-throughput screenings accumulated by pharmaceutical companies during the past decades [19], we decided to test the effect of each inhibitor at a single concentration (5 μM) on 4 CRC-SC lines bearing either wild-type or mutant KRAS, and two KRAS mutant CRC commercial cell lines, HCT116 and SW480. After 48h treatment most of the compounds tested displayed low or no efficacy on the CRC-SC lines (Figure 1A). A significant reduction of cell number below −1.5 x standard deviations (SD) from the overall mean viability was present, in all the cell lines, only for two drugs tested (Figure 1A). One of them, 5-iodotubercidin, is a multi-kinase inhibitor whereas Ro 31-8220 is a PKC inhibitor. Apart from one CRC-SC line, where the efficacy barely reached the −1.5 x SD threshold, Tyrphostin-9, a PDGFR inhibitor and Rottlerin, another PKC inhibitor that is more active on the δ iso-enzyme, were among the most effective drugs tested. Nonetheless, isolated inhibition of the pathways analyzed was not sufficient to efficiently and specifically interfere with CRC-SC survival and/or proliferation, with the possible exception of PKC, which is a broadly connected signaling hub [20]. Besides Rottlerin, several other chemicals exerted an inhibitory effect on CRC-SC viability but with a non-consistent pattern among the different cell lines, pointing primarily at EGFR, AKT, ERK and GSK3-β. In order to further evaluate the impact of the positive target hits on the viability of CRC-SCs or SW480 and HCT116 cell lines, we titrated down to 200 nM the compounds that in the initial screening led to a decrease in viability ≥ 1.5 x SD. The anti-proliferative activity of inhibitors of EGFR, GSK3-β and CaM kinase II was not maintained below 5 μM, irrespectively of KRAS mutation status. Conversely, a concentration-dependent effect was observed for at least one of the inhibitors acting against ERK, AKT, PDGFR and PKC (Figure 1B). Differently from what observed for CRC-SCs at doses lower than 5 μM, many of the selected compounds mantained their anti-proliferative effects on SW480 and HCT116 cell lines even if at lesser extent (Figure 1B), suggesting that inhibition of targeted signaling pathways could be effective in eliminating the non-stem compartment of the tumor.

### Multitarget inhibition by UCN-01 significantly impairs CRC-SC survival *in vitro*

**Figure 2:**
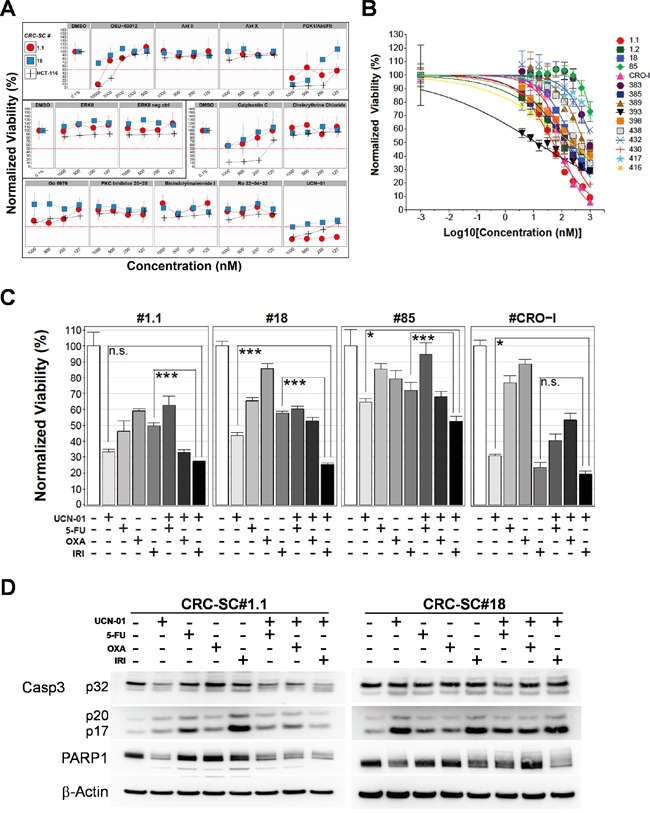
Treatment of CRC-SCs with drug analogues of positive hits identifies UCN-01 as an anti-CSC agent that enhances the effects of chemotherapy *in vitro* **A.** Titration of compounds analogue of drugs selected after screening with a kinase inhibitor library and resulting from dose-response tests in the low micromolar range. Drug names are reported on top of each plot and drugs sharing the same pathway targets are grouped in three boxes (AKT, ERK and PKC respectively). **B.** Dose-response curves of CRC-SCs after 72h of treatment with UCN-01. The mean±SD (n=3) and curve fitting lines are reported for each CRC-SC tested. **C.** Mean±SD (n=3) bar chart of 48h combinatorial treatment of UCN-01 and chemotherapeutic agents in 4 representative CRC-SC lines. Statistical comparison of UCN-01 alone versus UCN-01/irinotecan combination was done by means of Student's t test and Bonferroni correction and is indicated on top of the relative bars. **D.** Western blot analysis of Caspase-3 and PARP cleavage after 48h single-agent UCN-01 and chemotherapy or combined treatments in wild-type (#1.1) or KRAS mutant (#18) CRC-SC lines. Drug concentrations were 5-FU: 25 μM, oxaliplatin: 10 μM, irinotecan: 25 μM and UCN-01 500nM. Due to the different intensity of the signal, images of procaspase 3 (p32) and cleaved caspase 3 fragments (p20-p17) belong to different time exposure.

To confirm the relevance of potential targets emerging from kinase inhibitor screening, we tested the efficacy of drug analogues on HCT116 and two representative CRC-SC lines, i.e. #1.1 and #18, chosen based on their different KRAS and TP53 status. Although MEK inhibitors (U-0126 and PD98059) did not show efficacy in the previous screening, as an experimental control we tested an analogue ATP-competitive inhibitor of ERK1 and ERK2 which, as expected, did not produce significant effects in the cell lines tested similarly to its negative control (Figure 2A). A significant decrease of CRC-SC viability was observed in experiments performed using a set of PKC-inhibitors and four different AKT-inhibitors (Figure 2A). While a clear efficacy of the DAG-binding competitor calphostin-C was evident, single inhibition of AKT was not sufficient to cause death in CRC-SC lines, reinforcing the concept that CSC survival and/or proliferation is driven by concomitant activation of multiple pathways. In line with this observation, the dual-pathway inhibitor PDK1/AKT/FLT showed significant effects in both CRC-SC lines. Nonetheless we explored the possibility to inhibit AKT pathway through a Celecoxib-derived compound that lacks cyclooxygenase-2 (COX2) inhibitory activity and targets PDK1 (OSU-03012), without observing significant effects (Figure 2A). We also performed combinations of OSU-03012 with AKT-targeting compounds in order to reproduce the effects of the dual pathway inhibitor, but we could not observe synergistic effects (data not shown).

Among PKC inhibitors, we tested the staurosporine derivative UCN-01, which is a multi-kinase inhibitor that potently inhibits both AKT and PKC pathways (Figure 2A). HCT116 cell line resulted more sensitive than CRC-SC lines thus we decided to focus only on CRC-SCs for subsequent experiments. Since UCN-01 was effective on both CRC-SC lines tested (Figure 2A) we performed a dose-response curve assay on a wide panel of 15 CRC-SC lines (Figure 2B). After 72h of treatment, 11 of the cell lines showed a clear concentration-related inhibition of the proliferation with r^2^ above 0.58 and IC_50_ below 1 μM whereas 4 CRC-SCs showed resistance to the treatment (IC_50_ > 1μM, Table 2). We then tested the effect of UCN-01 in combination with irinotecan, oxaliplatin or 5-FU, drugs included in standard chemotherapy protocols for colon cancer. UCN-01 treatment was extremely effective as a single agent in two out of four CRC-SC lines. The combination of UCN-01 with irinotecan, but not with oxaliplatin or 5-FU, produced a significantly stronger effect than UCN-01 alone in the two KRAS mutant CRC-SC lines. Conversely, in the two KRAS wild-type CRC-SC lines, the effect of the UCN-01/irinotecan combination was mostly due to irinotecan in the CRC-SC line #CRO-I and to UCN-01 in the CRC-SC line #1.1 (Figure 2C).

**Table 2:** UCN-01 dose-response curve fitting data

CRC-SC #	IC50 (nM)	IC50 95% conf. int. (nM)	R square
*393*	12.78	4.477 to 36.45	0.6922
*1.1*	48.95	41.89 to 57.21	0.8979
*CRO-I*	91.59	79.94 to 104.9	0.8855
*430*	142.1	120.0 to 168.4	0.9592
*385*	143.2	123.1 to 166.7	0.94
*416*	199.6	132.0 to 301.8	0.8856
*1.2*	207.2	111.1 to 386.6	0.5836
*18*	251	215.0 to 293.0	0.8518
*398*	304.8	258.1 to 360.1	0.9439
*438*	436.9	383.5 to 497.7	0.9666
*389*	613.4	403.4 to 932.6	0.7345
*432*	1299	702.6 to 2403	0.6504
*85*	1560	1077 to 2261	0.381
*417*	9954	2472 to 40084	0.6524
383^*^	>1000		

* model convergence failed

Finally, in two representative CRC-SCs treated with either oxaliplatin, irinotecan or 5-FU, given alone or in combination with UCN-01, we show that the cytotoxicity induced by UCN-01 combined or not with irinotecan is mediated by activation of caspase 3 in CRC-SC line #1.1, as assessed by the decrease of the full-length caspase 3 (procaspase 3) and of its downstream target Poly-(ADP-ribose) polymerase (PARP) (Figure 2D). Activation of caspase 3 was less evident in the KRAS mutant CRC-SC line #18 even though the combined treatment strongly reduced PARP. To investigate the possible contribution of other mechanisms of cell death, i.e. authophagy, we evaluated the expression of autophagosomal marker LC3-II, which was not significantly modulated by treatment with UCN-01 in both CRC-SC lines (data not shown). The ability of UCN-01 to induce apoptosis alone or in combination with conventional drugs was evaluated by Annexin V/PI staining (Supplementary Figure S2). Treatment with UCN-01 alone or in combination with irinotecan increased the portion of Annexin V-positive cells compared to vehicle-treated cells, in both CRC-SC lines, irrespective of KRAS status. These results suggest that even in the KRAS mutant cell lines, UCN-01 alone or in combination with irinotecan is able to induce apoptosis at least in part mediated by a caspase 3-independent mechanism [21–24].

### UCN-01 potentiates the ability of irinotecan to reduce tumor growth

**Figure 3:**
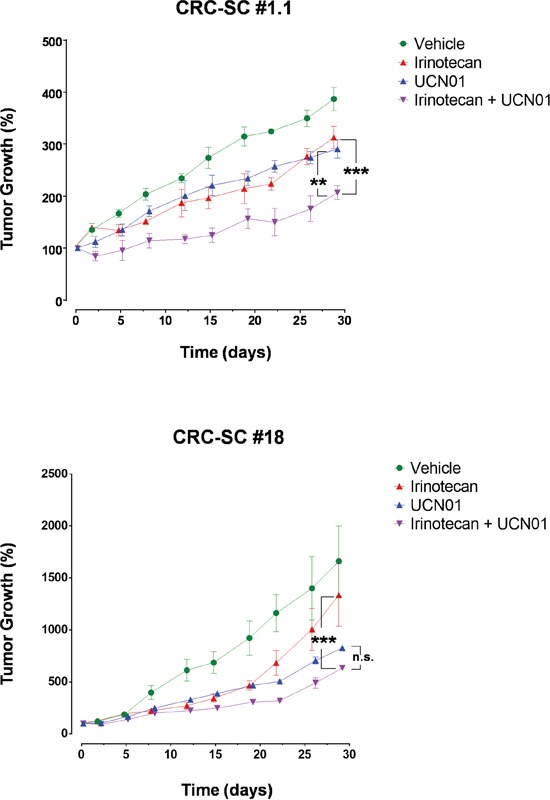
Treatment of CRC-SCs with UCN-01 enhances the effects of irinotecan *in vivo* Tumor volume increase curves in NSG mice xenografted with a KRAS wild-type (#1.1) or mutant (#18) CRC-SC treated by i.p. injection with either vehicle (DMSO), UCN-01 (5mg/Kg), irinotecan (10mg/Kg) or combined UCN-01 and irinotecan. Values are reported, for each cell line and drug tested, as the mean±SD of two independent experiments each comprising two biological replicates. Statistical comparison of UCN-01 or irinotecan alone versus irinotecan/UCN-01 combination is reported for the latest time point and was calculated by means of two-way ANOVA and Tukey's multiple comparisons test.

Subcutaneous (s.c.) injection of CRC-SC lines into immunodeficient mice results in the generation of tumors bearing the phenotypic and histologic features of parental human counterparts (Supplementary Figure S1). Therefore, we assayed the effect of UCN-01 either alone or in combination with irinotecan, on s.c. tumors generated by injection of either #1.1, the most sensitive CRC-SC in *in vitro* drug combination experiments, or #18 the CRC-SC line in which additive effect of the combination was the most apparent *in vitro*. Compared to vehicle-treated controls, UCN-01 alone reduced the growth rate of the tumors at a greater extent than irinotecan alone while, the combination of the two agents resulted in increased efficacy when compared to single treatments (Figure 3). Differently from what observed *in vitro*, CRC-SC line #18 showed a sensitivity to single agent UCN-01 at least similar, if not higher, than line #1.1 (Figure 3).

### Cytotoxic effects of UCN-01 involve Chk1 inhibition

**Figure 4:**
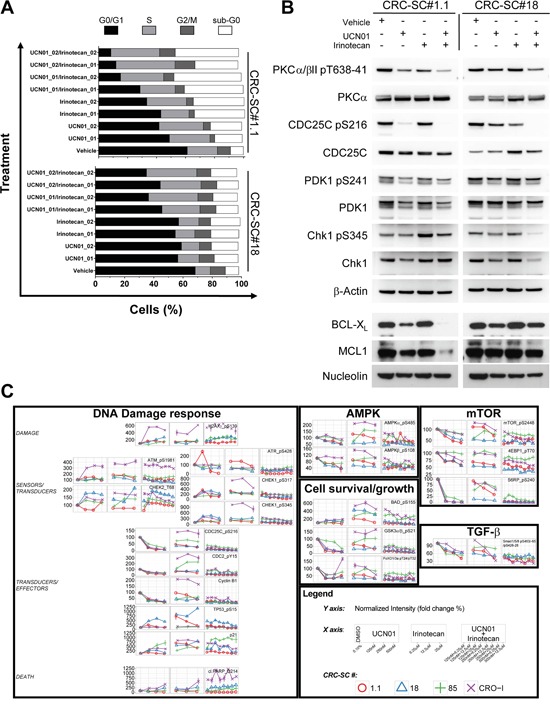
Combination of UCN-01 with irinotecan blocks CRC-SC replication by targeting the DNA damage pathway **A.** Cell cycle analysis of two representative CRC-SCs after 48h treatment with two different doses of UCN-01 (250 and 500nM, respectively 01 and 02) or irinotecan (12.5 and 25 μM, respectively 01 and 02) and their combinations. **B.** Western blot analysis of UCN-01 targets and BCL2 family proteins after 48h treatment with UCN-01, irinotecan or their combination (doses are 500nM and 25 μM, respectively). **C.** Time course plots of RPPA data obtained on 4 CRC-SCs after 24h treatment with UCN-01, irinotecan and their combinations at the indicated concentrations. Data are expressed in percent calculated over the vehicle control (DMSO 0.1%).

To further analyse the molecular mechanisms through which UCN-01 exterts its cytotoxic effects, we evaluated DNA cell content in CRC-SCs treated with UCN-01 alone or in combination with irinotecan. A consistent increase in the pre-G_0_ peak and the S phase was apparent in the cell lines tested and, particularly in line #1.1 where inhibition of *in vitro* cell growth was more evident. Pre-G_0_ peak increase indicates that induction of apoptosis significantly contributes to the cytotoxic effect of UCN-01 (Figure 4A).

Although UCN-01 is widely recognized as a broad-spectrum inhibitor of the PKC family of enzymes, it has also been demonstrated that one of its preferential targets is the checkpoint kinase Chk1, directly acting on the dual-specificity phosphatase CDC25C, as well as on PDK1, which acts upstream of AKT. In order to understand whether such signaling pathway contributes to the effect of UCN-01 on CRC-SCs, we evaluated the expression levels of total and phosphorylated PDK1 (pS241), PKCα/β II (pT638/41), CDC25C (pS216) and Chk1 (pS345) in two representative CRC-SC lines. Immunoblotting analysis demonstrated that UCN-01 alone or in combination with irinotecan affects the ATR-mediated phosphorylation of Chk1 and PKC directly blocking the activation of the Chk1 target CDC25C (Figure 4B), confirming the contribution of several targets in determining the response of CRC-SCs to UCN-01. PDK1 phosphorylation was slightly reduced in both cell lines by the combination of UCN-01 and irinotecan. Chk1 phosphorylation has been described to be a useful biomarker for monitoring inhibition of Chk1 activity, both *in vitro* and in clinical trials [25]. However, growing evidences show that, treatment with cytotoxic chemotherapeutic agents as well as Chk1 inhibitors, may result in a marked reduction of total and phosphorylated Chk1 [25]. Moreover, it has been demonstrated that inhibition of Chk1 activity paradoxically leads to the accumulation of its phosphorylated forms (pS317 and pS345) and that ATR catalyzes Chk1 phosphorylation under these conditions [26]. In line with these observations we found that combination of UCN-01 with irinotecan is able to significantly reduce both total and phospho-Chk1 (pS345) in the KRAS/TP53 mutant CRC-SC line #18. Conversely, levels of phosphorylated Chk1 (pS345) slightly increased in the KRAS/TP53 wild type CRC-SC line #1.1 (Figure 4B).

In line with the ability of UCN-01 to enhance the pro-apoptotic signals induced by irinotecan, the expression of anti-apoptotic proteins BCL-X_L_ and MCL1 were decreased by co-treatment with UCN-01 but only in the KRAS wt CRC-SC line (Figure 4B).

**Figure 5:**
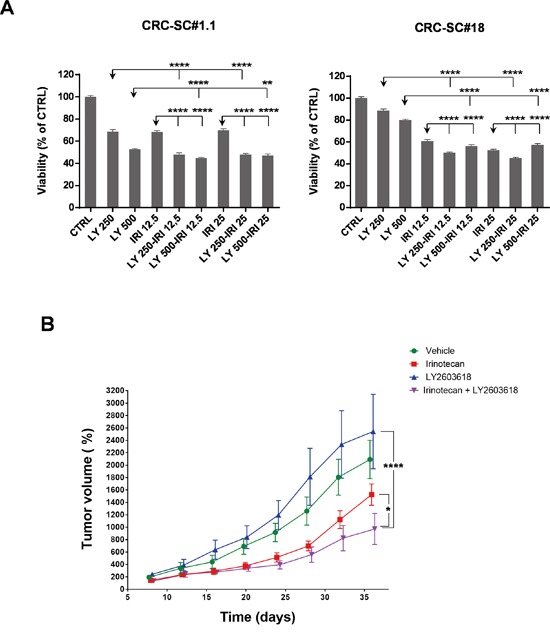
Treatment with the Chk1/2 inhibitor LY2603618 enhances the efficacy of irinotecan *in vitro* and *in vivo* **A.** Mean±SD (n=3) bar chart of 48h combinatorial treatment of LY2603618 (250 and 500 nM) and irinotecan (12.5 and 25 μM) in two representative CRC-SC lines. Statistical comparison of LY2603618 alone *versus* LY2603618/irinotecan combination was done by means of one way ANOVA and Tukey's multiple comparisons test, levels of significance are indicated on top of the relative bars. **B.** Mean±SD (n=2) of normalized tumor volumes (see Materials and Methods section) in NSG-mouse xenografts of the KRAS mutant CRC-SC line #18 after i.p. treatment with either vehicle (DMSO), LY2603618 (5mg/Kg), irinotecan (10mg/Kg) or combination of LY2603618 and irinotecan. Statistical comparison of LY2603618 or irinotecan alone versus irinotecan/LY2603618 combination for the latest time point was done by means of two-way repeated measures ANOVA and Tukey's multiple comparisons test.

In order to gain additional insights into the molecular mechanisms behind the enhanced cytotoxic effects of UCN-01/irinotecan combination, we studied the activation status of other proteins involved in DNA-damage, PI3K/mTOR, AMPK, MAPKs and TGF-β signaling pathways by RPPA. Phosphorylation status of the RPPA endpoints analysed in four CRC-SC lines showed heterogeneous patterns of pathway activation and no clear association with the sensitivity to single agents or combined treatment at specific timepoints and drug concentrations (Supplementary Figures S3 and S4). Nonetheless, dose-response analysis of the molecular changes caused by both single and combined treatments, demonstrated that UCN-01 might either reinforce or amplify the action of irinotecan on CRC-SCs by modulating diverse components of the DNA damage/Cell cycle checkpoint (Figure 4C and Supplementary Figures S3 and S4). These observations induced us to test the effects of a commercially available Chk1/2 inhibitor, i.e. LY2603618, either as a single agent or in combination with irinotecan on CRC-SCs. Concomitant treatment with the Chk1/2 inhibitor significantly increased the ability of irinotecan to inhibit *in vitro* cell proliferation of both KRAS wild type and mutant CRC-SC lines (Figure 5A). Moreover, combined treatment was effective in inhibiting *in vivo* tumor growth of KRAS/TP53 mutant CRC-SC line. (Figure 5B). The LY2603618 activity as a single agent or combined with irinotecan was evaluated by immunoblot showing a modulation of both total and phosphorylated Chk1 and its downstream effector Cyclin-dependent Kinase 1 (CDK1, also known as CDC2). LY2603618 did not potentiate the reduction of phosphorylated and total PDK1 or PKC levels induced by irinotecan (Supplementary Figure S5). Treatment with LY2603618, did not result in a marked activation of caspase 3 as assessed by immunoblot analysis (Supplementary Figure S5).

Finally, since Chk1 is the target of ATR kinase activity, we tested AZ20, a novel, potent and selective inhibitor of ATR kinase (IC50 5nM), on two CRC-SC lines using time/concentration conditions employed for UCN-01 and LY2603618. As shown in Supplementary Figure S6 AZ20 alone or in combination with irinotecan exerts cytotoxic effects similar, in CRC-SC line #18, or reduced, in CRC-SC line #1.1, compared to LY2603618.

Taken together these results confirm that concomitant inhibition of DNA damage and DNA replication pathways increases the efficacy of conventional chemotherapy on CRC-SCs.

## DISCUSSION

In recent years there has been a rapid increase in therapeutically available approaches for CRC and this, in turn, has led to an improvement of overall patients' survival. Irinotecan or oxaliplatin introduced into the clinics more than ten years ago, in association with 5-FU and folinic acid, remain the main therapeutic options for CRC patients in advanced stage even if response to these treatments is not always definitive, with a mean 5-year survival rate of about 40% [27, 28].

Recently characterized aberrant/hyperactive signaling pathways in CRC led to the understanding of molecular mechanisms behind initiation and progression of such neoplasm and allowed the identification of potential molecular targets and predictors for new therapies [29]. Protein kinases represent an important group of enzymes crucially involved in controlling survival and tumor progression signals and their inhibition is conventionally obtained by two main classes of compounds: monoclonal antibodies, acting mainly by competition with natural ligands on cell surface tyrosine kinase (TK) receptors, and small molecule inhibitors of natural or synthetic origin, acting also on intracellular enzymes/receptors. The use of these compounds has been applied to CRC treatment, in order to selectively inhibit tumor-associated activity of kinases such as EGFR, VEGFR, PDGFR, PI3K and mTOR [30]. Although targeted therapy has produced a significant increase in progression free survival of CRC patients, present clinical protocols are still ineffective in definitively eradicating the tumor in advanced cases [31]. The limited efficacy of currently available anti-cancer therapies has recently been linked to the possibility, supported by a growing body of evidence, that cancer is a stem cell disease. In particular, the recent identification of cell populations bearing stem cells characteristics in human tumors, including CRC, suggested that these might originate from a small fraction of tumor cells, referred to as “cancer stem cells” (CSC) [11, 12]. CSCs are characterized by self-renewal ability, multipotency and tumorigenicity in permissive hosts but also display low sensitivity to chemotherapy [32, 33] and radiotherapy [34] and are capable of initiate tumor growth in the original or in distant sites [18]. Therefore, CSCs are a target of choice for novel selective therapies aimed at a complete tumor eradication. The comprehension of CRC-SC behavior and, in particular, of the molecular mechanisms that regulate self-renewal, survival, apoptosis, proliferation and differentiation is of major interest to induce their efficient elimination and, in turn, a complete tumor regression. To this aim, we screened our CRC-SC lines with a library including 80 protein kinase inhibitors with characterized target specificity. This analysis led to the identification of critical pathways potentially involved in proliferation and survival of CRC-SC such as EGFR, AKT, MEK, PKC, PDGFR and GSK3-β. However, scaling down to sub-micromolar concentrations, most positive hits from the high-concentration screening failed in maintaining their efficacy. Treatment with compounds analogues of the drugs that kept efficacy at reduced doses demonstrated that, differently from all other molecules tested in this study, UCN-01 is able to efficiently induce cell death and to increase the cytotoxicity of irinotecan in CRC-SCs with diverse genetic background. UCN-01 is a staurosporine derivative that, differently from its parent molecule, displays significant activity against a handful of kinases. Initially identified as a PKC inhibitor, UCN-01 also targets Chk1 and PDK1 [35]. Notably, the efficacy of UCN-01 as an antineoplastic agent in combination with chemotherapy, has been evaluated in phase I and II clinical trials on solid tumors [36–39].

Although CSCs play an important role in CRC progression and growth, conventional treatment for CRC is required to eliminate the aberrantly proliferating cells that form the bulk of the tumor and is important also because inter-conversion between non-stem and stem status has been described [40, 41]. In our hands, UCN-01 affected the viability of CRC-SCs by increasing irinotecan cytotoxicity and inducing cell death. SN-38, the most active metabolite of irinotecan, is known to produce DNA damage by blocking topoisomerase I and triggering, by itself, a p53-dependent apoptotic response [42, 43]. Indeed, following irinotecan treatment, we observed decrease of full-lenght Caspase-3 and of its target PARP. Combination with UCN-01 further increased PARP cleavage and simultaneously reduced the levels of the anti-apoptotic proteins upregulated by PKCα [44, 45], BCL-X_L_ and MCL1, the latter only in the KRAS wt cell line, thus suggesting a potential mechanism for the additive effects of UCN-01 and irinotecan.

Another UCN-01 target, PDK1 is known to act upstream of the PI3K/AKT pathway promoting the expression of BCL-2 family proteins [46]. Here, UCN-01 treatment contributes to enhance apoptosis induced by irinotecan possibly through the marked reduction of PDK1 auto-phosphorylation (pS241). *In vivo* analysis of UCN-01 sensitivity in murine heterotopic xenograft models of CRC-SC growth, confirmed our previous *in vitro* observations, demonstrating that UCN-01/irinotecan combination significantly impairs tumor growth compared to chemotherapy alone. RPPA analysis of the molecular mechanisms underlying the enhanced cytotoxic effects of UCN-01/irinotecan combination showed that many components of the DNA damage/cell cycle checkpoint are affected. Particularly, even though the four CRC-SC lines showed heterogeneous patterns of pathway activation and no clear association with the sensitivity to single agents or combined treatment at specific timepoints and drug concentrations, we found a specific inhibition of Chk1 kinase and its main targets, i.e. CDC25C phosphatase and CDK1 (CDC2). Thus, we hypothesize that the impairment of CRC-SC growth both *in vitro* and *in vivo* is associated with Chk1 targeting. Chk1 phosphorylates CDC25C in response to DNA damage and following replication fork stalling, thus preventing cell cycle advancement and allowing repair of DNA damage induced by chemotherapy in CSCs [47, 48]. Accordingly, it has been demonstrated in a commercial CRC line model that irinotecan/UCN-01 treatment is effective only in the absence of TP53 or CDKN1A (p21) or both [43]. In the context of CSCs, the additive effects showed by UCN-01/irinotecan combination could result from the interference with CSC responses to chemotherapy-induced genotoxic stress irrespective of TP53 status. Nonetheless, co-treatment with the selective Chk1/2 inhibitor LY2603618, was able to potentiate irinotecan efficacy *in vitro* and in our mouse model of CRC growth, confirming a pivotal role of Chk1 in this context.

Altogether our data obtained on a CRC-SC-based model system indicate that Chk1 inhibition potentiates the antitumor activity of standard chemotherapeutic agents and potentially may address resistance to them.

## MATERIALS AND METHODS

### CRC and CRC-SC lines

HCT116 and SW480 CRC cell lines were purchased from American Type Culture Collection (ATCC) and cultivated in the recommended media (see www.atcc.org for details).

CRC samples were obtained from Sant'Andrea Hospital (Rome) upon patients' informed consent, the procedure was approved by the local ethical committee. CRC-SCs were isolated as previously described [12]. Briefly, tumor samples were subjected to mechanical and enzymatic dissociation using type II collagenase (Gibco Invitrogen Inc., BRL, Rockville, MD) and DNase I (Roche, Mannheim, Germany). The resulting cancer cells were cultured in a serum-free medium supplemented with 20 ng/ml EGF and 10 ng/ml FGF-2 (PeproTech, Rocky Hill, NY). The expression of the stem cell marker CD133 and epithelial marker Ber-Ep4 have been evaluated by flow cytometry using the following antibodies: anti-CD133-PE (clone AC133/1, mouse IgG_1_, MiltenyiBiotec Inc., Bergisch Gladbach, Germany), anti-Epithelial Antigen-FITC (clone Ber-Ep4, mouse IgG_1_, DakoCytomation, Denmark) or isotype-matched control antibodies. Samples were analyzed with FACSCanto flow cytometer (Becton Dickinson, San Jose, CA) and data were analyzed with FACS Diva software (Becton Dickinson).

The ability to reproduce the original tumor has been evaluated by subcutaneous (s.c.) injection of CRC-SCs in immunodeficient NOD/SCID (Harlan Laboratories Italia, San Pietro al Natisone, Italy) or NOD/SCID IL2-Rγ-(NSG) mice (Charles River Italia, Calco, Italy). Tumors were removed, fixed in 10% neutral buffered formalin solution (Sigma Aldrich Inc., Saint Louis, MO) and paraffin embedded for histologic analysis. Supplementary Table S1 contains a list of the clinical features of the patients from which CRC-SCs were derived and Supplementary Figure S1 shows the paired comparison of H&E stainings obtained on CRC-SC-derived s.c. xenografts versus their original patient's tumor.

### CRC-SC line authentication and genetic characterization

CRC-SC lines were validated by Short Tandem Repeat (STR) DNA fingerprinting. Nine highly polymorphic STR loci plus amelogenin (Cell ID™ System, Promega Inc., Madison, WI) were used. Detection of amplified fragments was obtained by ABI PRISM 3100 Genetic Analyzer and data analysis was performed by GeneMapper^®^ software, version 4.0 (Biological Bank and Cell Factory, National Institute for Cancer Research, IST, Genoa, Italy). For all CRC-SC lines, profiles were compared against public databases to confirm authenticity.

Single-point mutations and small insertions-deletions in CRC-SCs were assessed by targeted DNA resequencing, focusing on 17 genes known to be frequently mutated in colon cancer and to play an important role in targeted therapy or prognosis. An amplicon-based custom panel was developed by using the Illumina Design-Studio software. The library for sequencing was prepared with the Truseq Custom Amplicon Kit following basically the manufacturer's instructions. Sequencing was performed on a MiSeq instrument.

Sequence FASTQ files were generated and analyzed through the MiSeq Reporter pipeline from Illumina. The analysis pipeline employs the Banded Smith Waterman algorithm 2.5 for read mapping and the Somatic Variant Caller (v3.5) for variant calling. Annotation of variants was carried out with Illumina Variant Studio software. Further in-house scripts were employed for mutation filtering and clustering, in particular with a minimum coverage of 100x, allele frequency of at least 5% and excluding all known SNPs with MAF >=5%. Oncoprints were generated with the integration of R scripts available at https://github.com/dakl/oncoprint.

### Drug cytotoxicity experiments

For cytotoxicity analysis CRC-SCs were mechanically dissociated and plated at a density of 2.5×10^4^ cells/ml, in triplicate, in a 96-well plate. 5-FU, Oxaliplatin and UCN-01 were purchased from Sigma, irinotecan and the small molecule kinase inhibitor library were from Enzo Life Sciences/Biomol (Farmingdale, NY, http://www.enzolifesciences.com/BML-2832/kinase-inhibitor-library). A list of the compounds used for the library screening is available as Supplementary Table S2. ERK inhibitor and its negative control (ERK inhibitor FR180204 II, ERK inhibitor II-Negative control), Akt and PKC inhibitors and the multiple inhibitor (Akt inhibitor II and X, PKC inhibitor Set, PDK1/Akt/Flt Dual Pathway Inhibitor) were from Calbiochem (Merck KGaA, Darmstadt, Germany), OSU-03012 and LY2603618 were from Selleck Chemicals (Houston, TX, USA). Compounds were dissolved in DMSO and added 16 hours after cell plating. ATP levels were measured using the CellTiter-Glo™ (Promega Inc) as per the manufacturer's instructions. The mean of the raw luminescence values (L_D_) from triplicate wells treated with vehicle alone (DMSO 0.2%, mL_C_), was used as reference to calculate percent viability from wells treated with drugs (V_D_), using the following formula: V_D_=(L_D_/mL_C_)*100.

### Cell cycle assay

For cell cycle analysis, 2×10^5^ CRC-SCs were plated in 6-well microtiter plates. After 16h, cells were treated with UCN-01 (250-500 nM) and irinotecan (12.5-25 μM) alone or in combination, after either at 24 or 48h., CRC-SCs were resuspended in Nicoletti's buffer containing propidium iodide 50 μg/mL [49].

Samples were analyzed using a FACSCanto flow cytometer (Becton Dickinson).

### Western blotting

Total protein content was extracted from cells using RIPA buffer (20 mMTris/HCl pH 7.2, 200 mMNaCl, 1% NP40) and Protease Inhibitor Cocktail and Phosphatase Inhibitor Cocktails II and III (Sigma-Aldrich). Samples were resolved in SDS-PAGE gels (NuPage 4-12% bis-tris Gel, Invitrogen). Protein expression was analyzed by standard western blot procedure using anti-Caspase 3 (Cell Signaling Technology, Danvers, MA, USA), anti-MCL1, anti-BCL-XL (both from Santa Cruz Biotechnology, Dallas, TX, USA), anti-PDK1, anti–PDK1_pS241, anti-PKCα/βII_pT638-41, anti Chk1 and anti-Chk1_pS345 (all from Cell Signaling Technology). The anti-nucleolin (Santa Cruz Biotechnology) or anti-β actin (Sigma Aldrich) antibodies were used as loading controls.

### CRC xenograft mouse models

Cells were resuspended in cold PBS and the suspension mixed with an equal volume of cold Matrigel (Becton Dickinson) at a final cell concentration of 5×10^6^ cells/mL. Mice were injected subcutaneously with 0.2 mL of the cell/Matrigel suspension. Treatments were initiated when the xenografts reached 10-13 mm in mean diameter, a size at which any change can be readily detected by caliper. Mice were treated for three weeks with 10mg/Kg irinotecan once a week on the first day and/or with 5mg/Kg UCN-01 (or with 5mg/Kg LY2603618) for 5 days a week. Treated mice were maintained up to 3 weeks without any further treatment, except for measurement of tumor mass, then sacrificed by cervical dislocation. Controls mice received an equal volume of saline/15% DMSO that was injected intraperitoneally (i.p.). Tumor growth percentage was calculated by normalizing over initial tumor volume. Animal experiments were performed in accordance with relevant institutional and national regulations.

### Reverse-phase protein microarrays

RPPAs were performed as previously described [50, 51]. Briefly, on day 0, CRC-SCs were enzymatically dissociated (TrypLE Select, Life Technologies Corporation, Carlsbad, CA), counted and plated into 24-well microtiter plates at a density of 5×10^5^ cells per well. The following day, CRC-SCs were treated with UCN-01 (125-250-500 nM) and irinotecan (6.25-12.5-25 μM) alone or in combination at all doses and cells were lysed either at day 2 (24h) or day 3 (48h). Cell lysates were diluted with 2X Tris-Glycine SDS Sample Buffer (Life Technologies) and final 2.5% TCEP (Thermo-Scientific, Whaltman, MA) prior to printing on nitrocellulose slides (Grace Bio-Labs, Bend, OR, USA) and were spotted in technical triplicates with the Aushon 2470 contact pin arrayer (AushonBioSystems Inc., Billerica, MA), in neat and 1:4 dilution pairs. Positive and negative control lysates were printed on every slide in a ten-point two-fold dilution curve. A subset of the printed slides were stained with by Sypro Ruby Blot Stain (Life Technologies) for assessment of total protein concentration. After incubation for 2 hours with I-Block (Life Technologies), array staining with antibodies was carried out on an automated slide stainer (Autostainer CSA kit, DAKO, Carpinteria, CA) using a biotin-avidin amplification system per the manufacturer's instructions (CSA kit, DAKO). Biotinylated secondary antibody was either goat anti-rabbit IgG H+L (Vector Labs, Burlingame, CA) or anti-mouse Ig (from CSA kit, DAKO). Streptavidin-conjugated IRDye680LT^®^ (LI-COR Biosciences, Lincoln, NE, USA) was used as a final signal generating step. Stained slides were scanned on a Tecan Power Scanner (Männedorf, Switzerland) equipped with a customized emission filter to increase efficiency in collection of IRDye680LT^®^ fluorescence. Image analysis for spot recognition, quantification and normalization was carried out using MicroVigene 5.1 software (VigeneTech Inc., Carlisle, MA, USA). Time course plots for all conditions and endpoints analysed by RPPA have been included in Supplementary Figure S2, after normalization over vehicle controls. All antibodies used for RPPA analysis are listed in Supplementary Table S3.

### Statistical analysis

For RPPA data analysis, unsupervised hierarchical clustering (Euclidean distance, Ward.D2 method) was performed on standardized data, by means of the package ‘gplots’ of the “R” software [52]. Plots in the manuscript were generated using ‘ggplot2′ package of ‘R’ while GraphPad Prism version 4.00 for Windows (GraphPad Software, La Jolla California USA, www.graphpad.com) was used for plots and non-linear fitting of UCN-01 dose-response experiments [4-parameter logistic (4PL) model: log(inhibitor) *vs* normalized response with variable slope]. Statistical comparison of Chk1 inhibitors alone *versus* combination with irinotecan was done by GraphPad Prism. Statistical significance was accepted for p values lower than 0.05 and astersiks reported in the plots indicate the level of significance as follows: * p<0.05, ** p<0.01, *** p<0.001 and **** p<0.0001.

## SUPPLEMENTARY FIGURES AND TABLES






