# Supplementary material for: UCN-01 enhances cytotoxicity of irinotecan in colorectal cancer stem-like cells by impairing DNA damage response
Source: Oncotarget. 2016 Jun 6;7(28):44113–28. doi: 10.18632/oncotarget.9859 (PMC5190083; doi:10.18632/oncotarget.9859)
Supplement: Supplementary file 1 [file oncotarget-07-44113-s001.pdf]

UCN-01 enhances cytotoxicity of irinotecan in colorectal cancer stem-like cells by impairing DNA damage response

SUPPLEMENTARY FIGURES AND TABLES

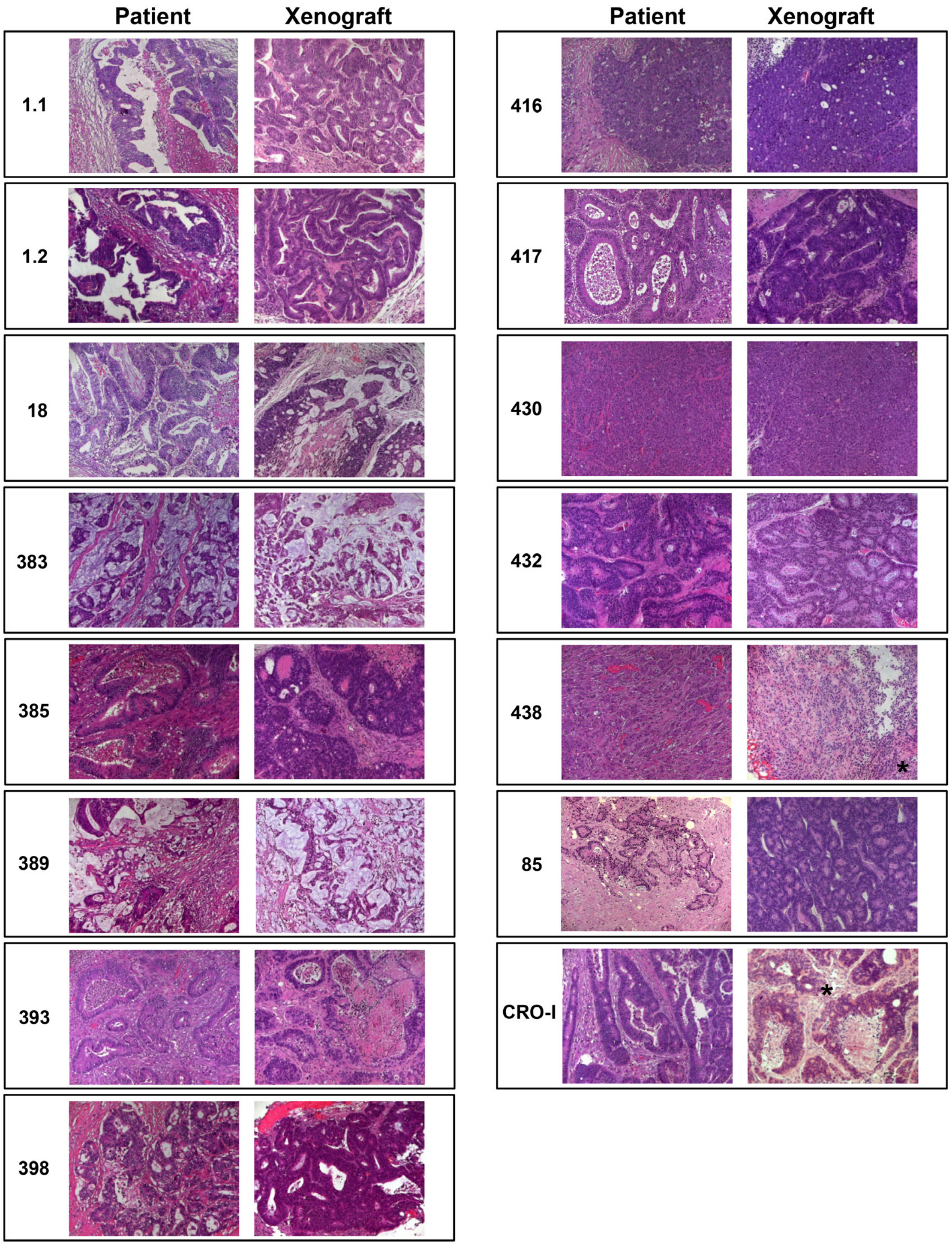

Supplementary Figure S1: H&E staining of paraffin embedded (*frozen) sections of patient tumors and xenografts generated by injection of the corresponding CRC-SC lines.

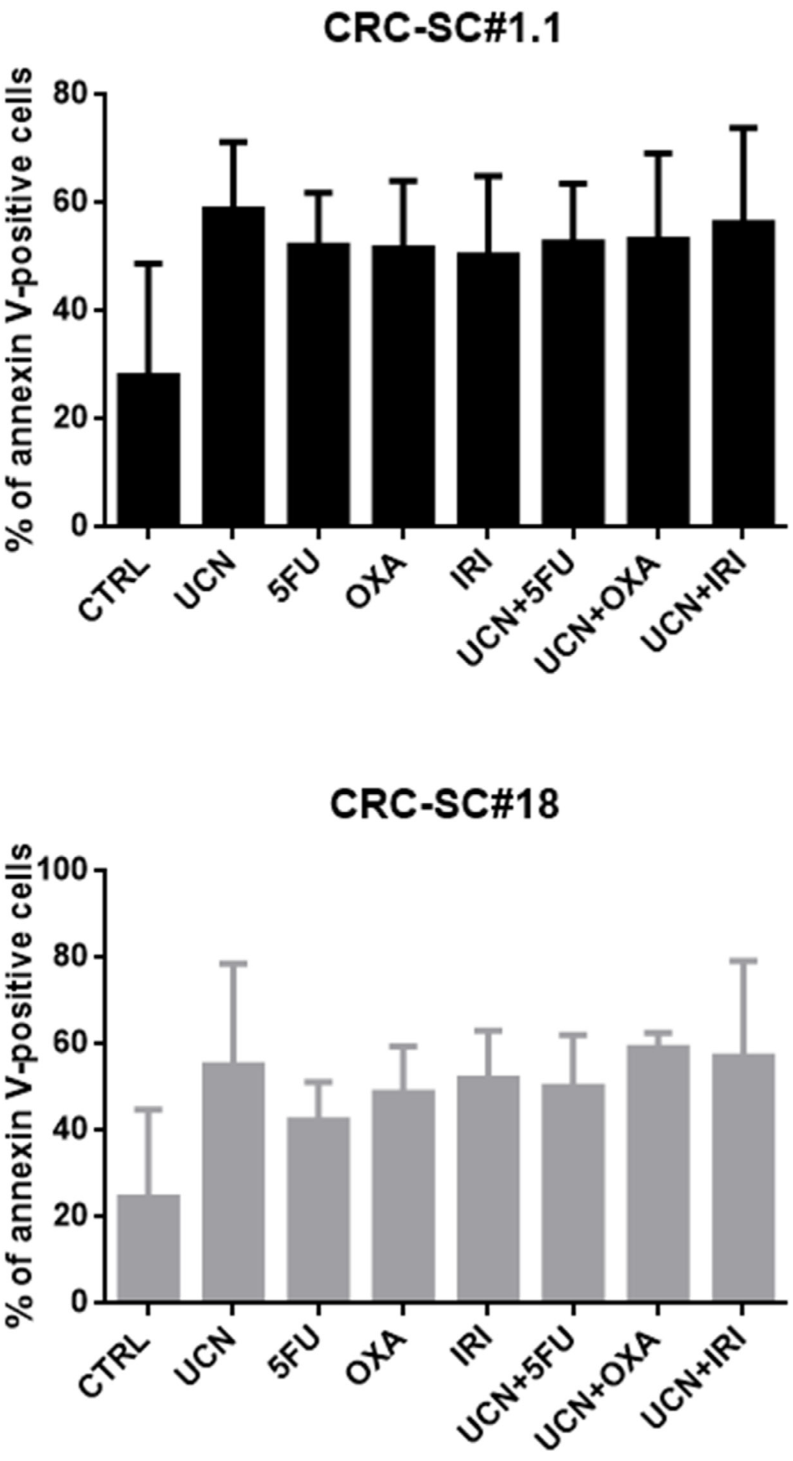

Supplementary Figure S2: Annexin V/PI (Annexin V-FITC Apoptosis Detection kit, Bender MedSystems GmbH, Vienna, Austria) binding to wild-type (#1.1) or KRAS mutant (#18) CRC-SC lines by FACS analysis 48h after treatment with UCN-01 (250 nM), irinotecan (25 µM), oxaliplatin (10 µM) and 5-FU (25 µM) alone or in combination. Statistical comparison of the combination versus the single treatment did not reach statistical significance.

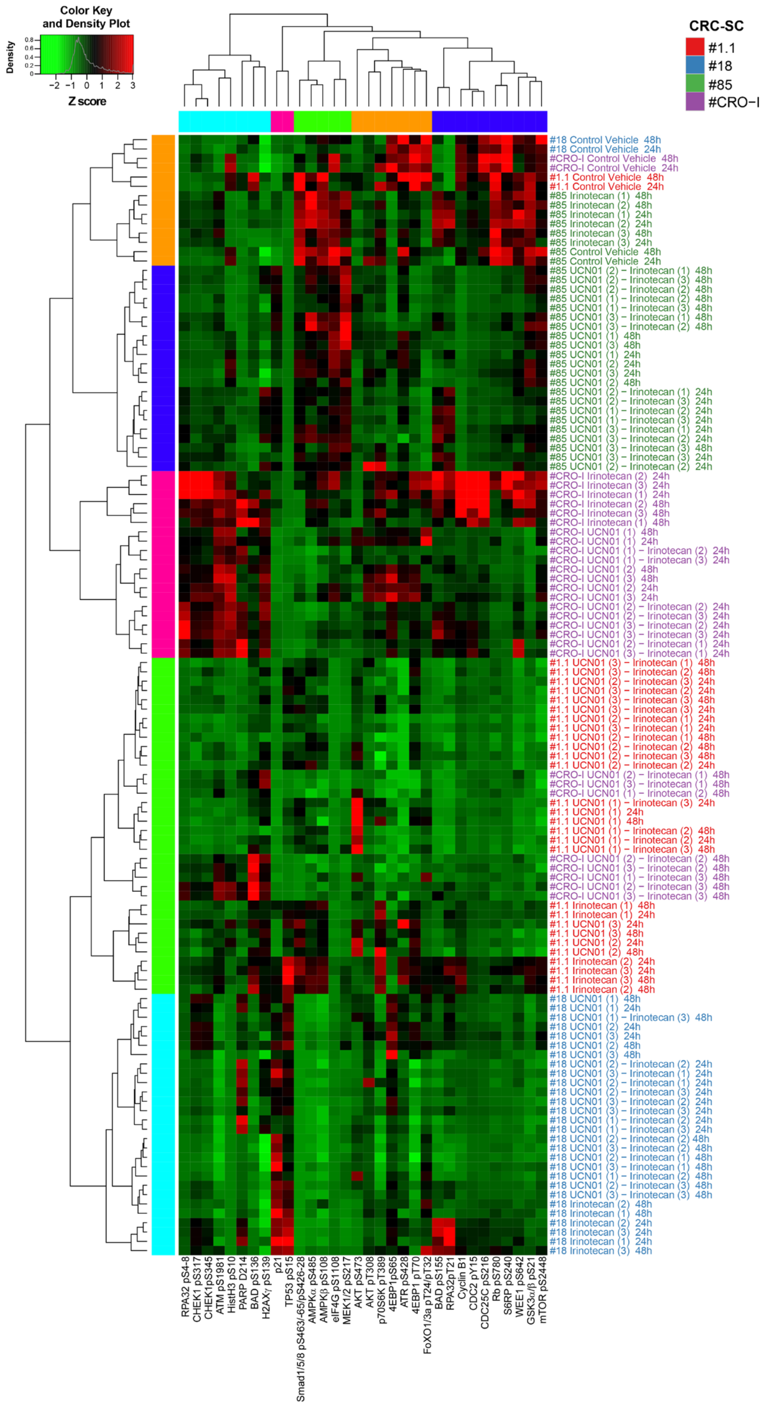

Supplementary Figure S3: Hierarchical clustering of RPPA data obtained on 4 CRC-SCs treated for either 24 or 48 h with three doses of irinotecan (25, 12.5 and 6.25 µM, respectively 1, 2 and 3) or UCN-01 (500, 250 and 125 nM, respectively 1, 2 and 3) and their combinations.

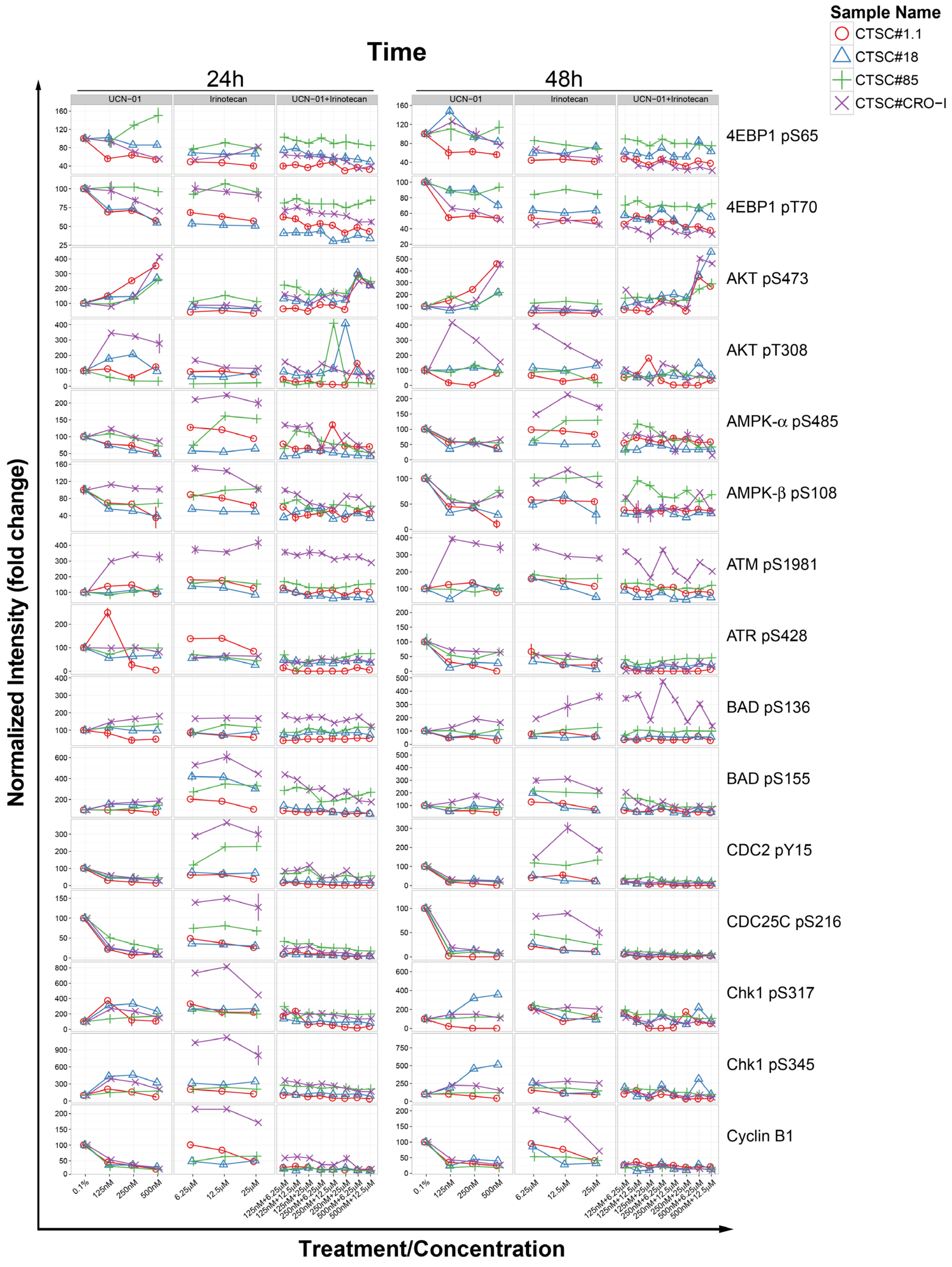
Sample Name
CTSC#1.1
CTSC#18
CTSC#85
CTSC#CRO−I
Time
24h
48h
UCN−01
Irinotecan
UCN−01+Irinotecan
4EBP1 pS65
4EBP1 pT70
AKT pS473
AKT pT308
AMPK-α pS485
AMPK-β pS108
ATM pS1981
ATR pS428
BAD pS136
BAD pS155
CDC2 pY15
CDC25C pS216
Chk1 pS317
Chk1 pS345
Cyclin B1
Normalized Intensity (fold change)
Treatment/Concentration
0.1%
125nM
250nM
500nM
6.25uM
12.5uM
25uM
125nM+6.25uM
125nM+12.5uM
125nM+25uM
250nM+6.25uM
250nM+12.5uM
250nM+25uM
500nM+6.25uM
500nM+12.5uM

(*Continued*)

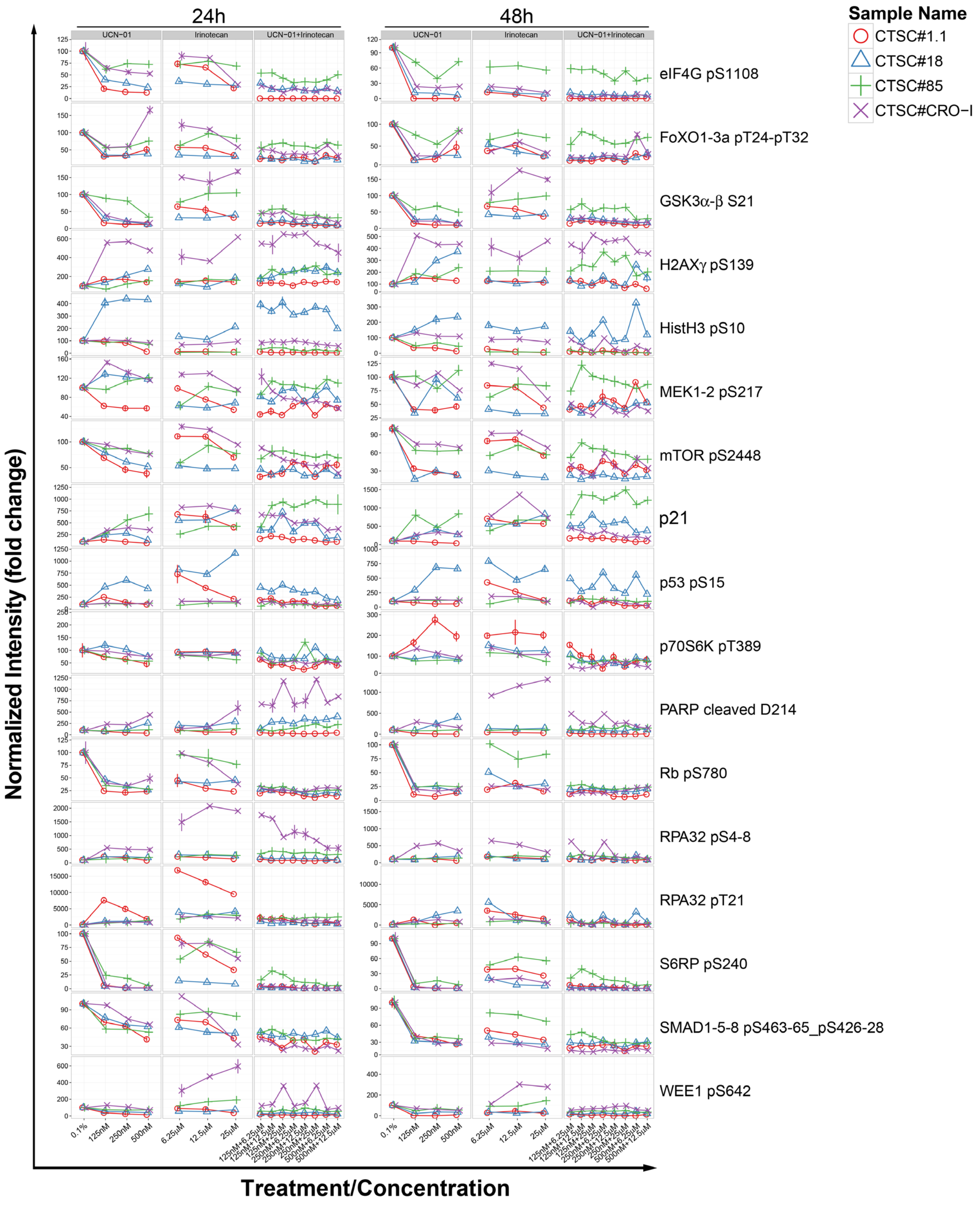

Supplementary Figure S4: Dose- and time-dependent plots of all protein and phospho-proteins measured by RPPA in 4 CRC-SCs after treatment with UCN-01 and Irinotecan alone or their combination at the indicated concentrations and time points. Data are expressed in percent calculated over the vehicle control (DMSO 0.1%).

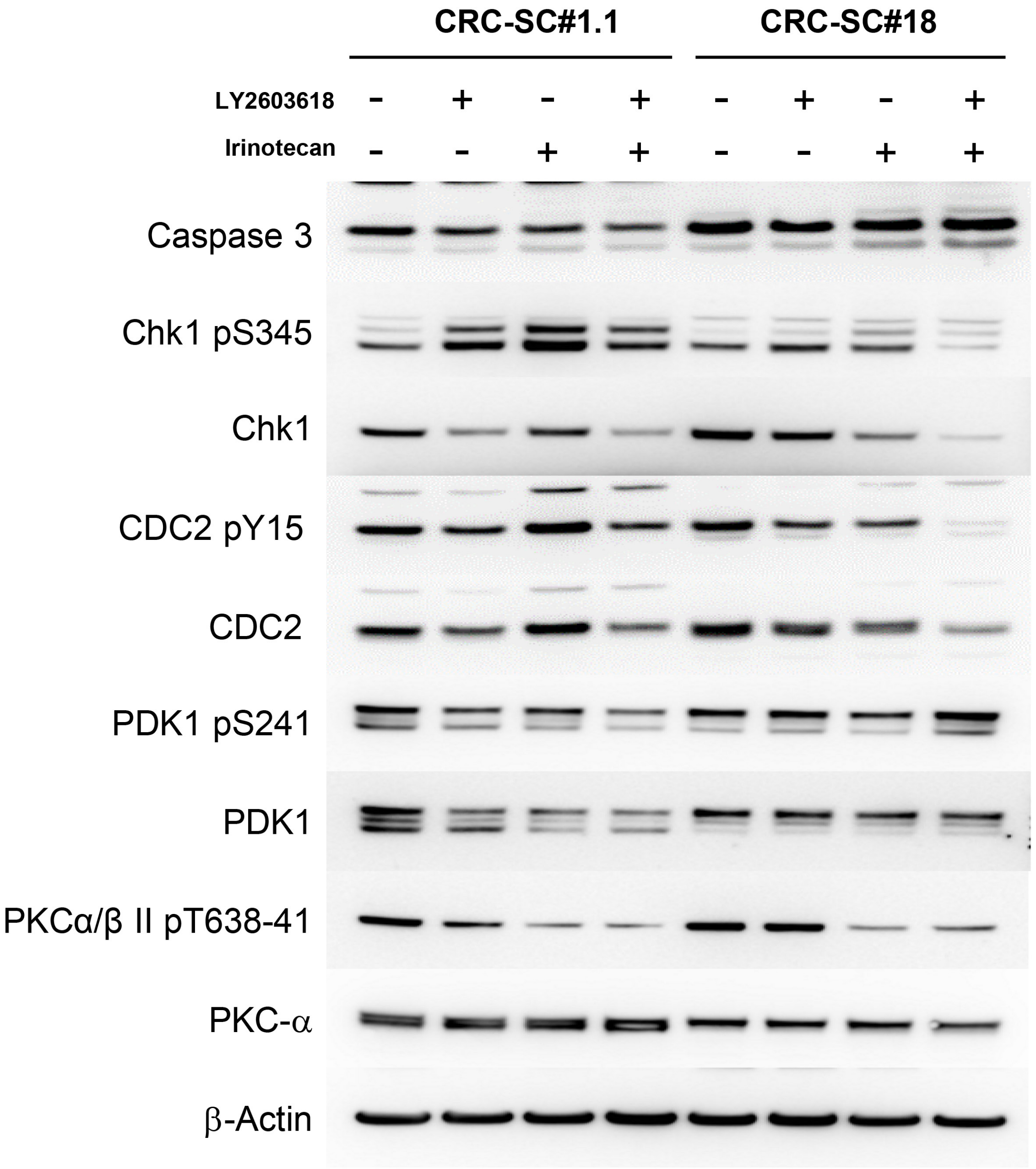

Supplementary Figure S5: Western blot analysis of full length Caspase-3, phospho-Chk1/total Chk1, phospho-CDC2/total CDC2, phospho-PDK1/total PDK1 and phospho-PKCα-β II/total PKCα after 48h of treatment with LY2603618 and Irinotecan or combined treatments in wild-type (#1.1) or KRAS mutant (#18) CRC-SC lines. Drug concentrations were 25µM for irinotecan and 500nM for LY2603618. Actin beta was used as loading control.

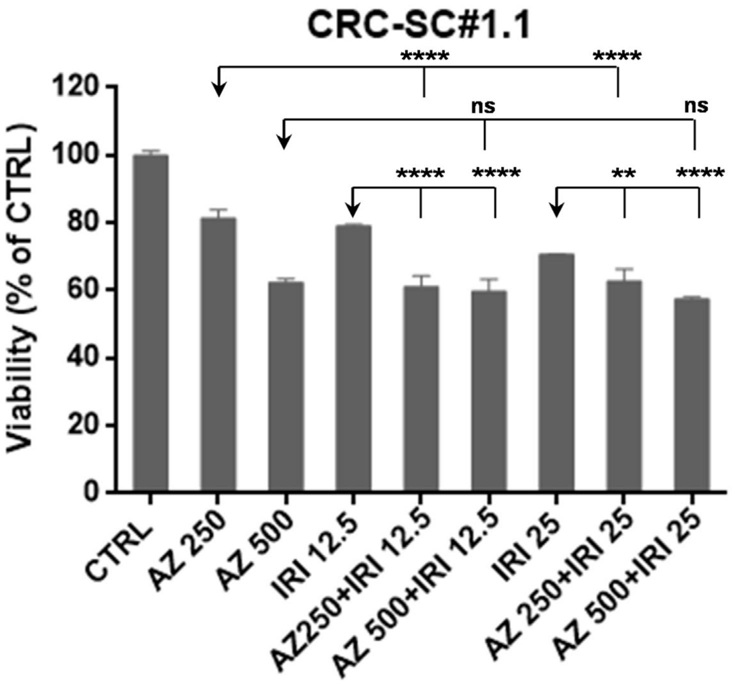

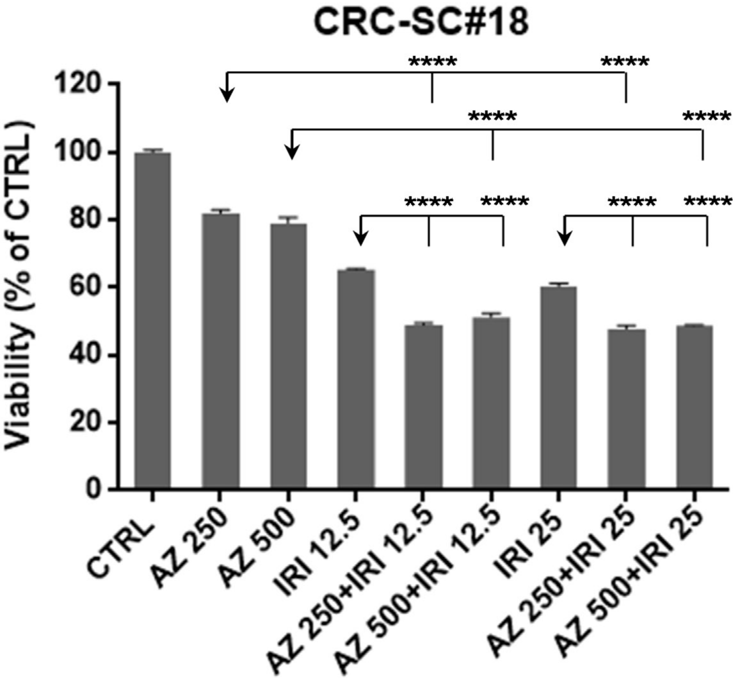

Supplementary Figure S6: Mean±SD (n=3) bar chart of 48h combinatorial treatment with AZ20 (250 or 500 nM) and irinotecan (12.5 or 25 µM) in the two representative CRC-SC lines #1.1 and #18. Statistical comparison of the combinations versus the single treatments was performed by ANOVA and Tukey's test. Levels of significance are indicated for each comparison.

Supplementary Table S1: List of patient characteristics and phenotypic features of correspondent CRC-SCs

PATIENTS							CRC-SCs	
CASE#	AGE	SEX	SITE	GRADE	pTNM	Dukes	CD133 (%)	Ber-EP4 (%)
1.1	68	M	Left	G3	IIIB	C	89.4	99.3
1.2	68	M	Left	G2	IIIB	C	92	98.6
18	66	F	Right	G2	IIA	B	47.6	83
CRO	63	F	Right	G2	IVA	D	88.3	96
85	64	M	Right	G2	IIA	B	37.7	89
383	80	M	Left	G3	IIIC	C	84	98.1
385	51	F	Left	G2	IIA	B	81.9	96.1
389	76	M	Right	G2	IIIC	C	49	82.8
393	57	M	Right	G3	IIIC	C	85.3	98.8
398	46	F	Left	G2	IIIB	C	81.1	86.5
416	82	M	Right	G3	IIIC	C	28.2	97.1
417	70	F	Right	G2	IIA	B	23.2	93.4
430	68	F	Right	G3	IIIB	C	98.1	99.8
432	49	M	Left	G3	IIIB	C	24.7	96.3
438	85	F	Right	G3	IIIC	C	31.3	95.9

Supplementary Table S2: List of drugs used for kinase inhibitor screening

See Supplementary File 1

Supplementary Table S3: List of antibodies used for RPPA analysis

See Supplementary File 2
